# Review of the Global Solar UV Index 2015 Workshop Report

**DOI:** 10.1097/HP.0000000000000742

**Published:** 2017-12-04

**Authors:** Peter Gies, Emilie van Deventer, Adèle C. Green, Craig Sinclair, Rick Tinker

**Affiliations:** *Australian Radiation Protection and Nuclear Safety Agency, 619 Lower Plenty Road, Yallambie, Victoria, Australia; †Radiation Programme, Department of Public Health, Environmental and Social Determinants of Health, World Health Organization, Geneva, Switzerland; ‡International Commission on Non-Ionizing Radiation Protection, 85764 Oberschleissheim, Germany; QIMR Berghofer Medical Research Institute 300 Herston Road, Herston, Queensland, Australia and CRUK Manchester Institute, University of Manchester, Wilmslow Road, Manchester, UK; §Cancer Council Victoria, 615 St Kilda Road, Melbourne, Victoria, Australia.

**Keywords:** radiation risk, reviews, ultraviolet radiation, World Health Organization

## Abstract

The Global Solar UV Index was developed as an easy-to-understand measure of the amount of biologically-effective ambient solar ultraviolet radiation (UVR) at different locations on the earth’s surface. Over the past few years, questions have been raised about the global applicability of the UV Index, about the evidence base for exposure risk thresholds and related protective measures, and about whether the overall impact of the UV Index could be improved with modifications. An international workshop was organized by several organizations, including the World Health Organization, to assess if current evidence was sufficiently strong to modify the UV Index and to discuss different ways it might be improved in order to influence sun-protective behavior. While some animal research suggests there may be no threshold effect, the relative importance of sub-erythemal doses of sunlight in causing skin cancer in humans remains unknown. Evidence suggests that regular use of sunscreen can prevent skin cancer and that sunglasses are an effective method of protecting the eyes from solar UVR. The UV Index as a risk communication tool continues to be useful for raising awareness and to support sun-protection behavior. Although there was agreement that guidance on the use of the UV Index could be improved, the workshop participants identified that strong health outcome-based human evidence would be needed as the basis for a revision. For the UV Index to be relevant in as many countries as possible, it should continue to be adapted to suit local conditions.

## INTRODUCTION

Excess Ultraviolet radiation (UVR) from sun exposure can lead to sunburn and importantly to chronic sun-induced skin damage, such as photoaging and skin cancer in the long term, and to ocular disorders including pterygia and cataracts. To meet the need for a standardized way to measure the intensity of UVR at a given location and time of day, both to serve as a public health risk awareness tool and to support studies of UVR-related disease, the Global Solar Ultraviolet Index was developed (UV Index or UVI). The UV Index is a unitless measure describing the levels of UVR reaching the earth’s surface at any given time and is a simple and informative indicator for the general public of the health risk of UVR exposure. The UV Index and associated guidance for its use have been developed by the World Health Organization (WHO), the World Meteorological Organization (WMO), the United Nations Environment Programme (UNEP), and the International Commission on Non-Ionizing Radiation Protection (ICNIRP).

First introduced in 1995, a revised version of the UV Index was published in 2002 to improve its utility as a public awareness tool on the potential harms of UVR and to alert people of the need for sun protection measures (WHO 2002). Since then, there has been increased attention paid to gaps in scientific knowledge related to the role of UVR in disease (e.g., cancers) and health (e.g., vitamin D production), to the effectiveness of the UV Index as a public awareness tool in encouraging sun-protective behavior, and to the applicability of the UV Index to all areas of the globe. In order to review the current scientific evidence around these questions and to ask whether sufficient new information was available to merit a revision of the UV Index, a workshop was held in association with the 3rd International UV and Skin Cancer Prevention Conference in Melbourne, Australia, in December 2015. The workshop was organized by WHO, ICNIRP, and by the two Australian WHO Collaborating Centres on UVR: the Australian Radiation Protection and Nuclear Safety Agency and the Cancer Council Victoria. This paper is a summary of that meeting in the context of recent scientific literature.

In 2009, the International Agency for Research on Cancer (IARC) classified ultraviolet radiation (UVR) of wavelengths 100 to 400 nm as carcinogenic to humans (IARC 2012). The range 100 to 400 nm encompasses UVC (100 to 280 nm), UVB (280 to 315 nm), and UVA (315 to 400 nm) (ICNIRP 2004).

### The UV Index as a predictor of UV-related health risks

The UV Index, which can be measured or calculated, is a standardized way to represent the amount of UVR reaching the earth at a particular time and location.

The equation to derive the UV Index is given by (WHO 2002):


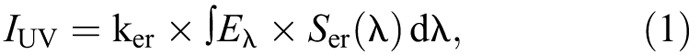


where *I*_UV_ is the UV Index, either measured or calculated; *E*_*λ*_ is the solar spectral intensity, either measured or calculated at wavelength λ; and *S*_er_ (*λ*) is the erythemal effectiveness of the CIE at wavelength λ (CIE 1998). By using the erythemal effectiveness, the UV Index takes account of the biological effects of the incident solar UVR to determine the potential hazard to the skin of people exposed to the sun.

Forecasts of the UV Index are broadcast in many countries to inform the public in advance so that risk from UVR exposure can be managed. Most commonly, these UVI predictions are created by modeling the UV irradiance accounting for relevant atmospheric parameters (total ozone, aerosol optical properties, and cloud cover). The predictive models in use vary in their complexity and accuracy, but all are dependent on good knowledge of the actual atmospheric parameters, which is often lacking (Dochain et al. 2000).

Direct UV Index measurements using spectroradiometers or broadband detectors can be used to support and evaluate predictive models. Worldwide, a large number of institutions measure the UV Index using a wide range of measurement systems. The sensitivity of these instruments is high in the UVB region, similar to the action spectrum for erythema, so their measurements are convertible to units of erythemal irradiance. Available instruments to measure the UV Index vary in their reliability, accuracy, and cost effectiveness, and calibration and quality control are required.

Current guidance on usage of the UV Index for risk prediction extends to values from 1 to 11+ and establishes five categories of exposure: “low” (<3), “moderate” (3 to <6), “high” (6 to <8), “very high” (8 to <11), and “extreme” (11 or greater). These exposure categories are further simplified into three groups for recommendations on protective measures. Factors that affect the amount of solar UVR reaching the earth’s surface (and hence the UV Index) include season, time of day, geographical location, environment (altitude, latitude, and reflective surfaces), atmospheric ozone levels, and cloud scatter of UVR.

As the UV Index is based on the reference action spectrum for UV-induced erythema on human skin, its primary role is as a predictor of skin damage and is less directly predictive of UV effects on the eyes or immune system. The individual health risk from UVR at any given value on the UVI scale depends on personal factors including duration of exposure, skin type, age, genetic inheritance, and use of protective measures.

#### Skin cancers

Fair-skinned people are most susceptible to skin cancer because they lack the protective melanin pigment of darker skin (Norval et al. 2014). There are three main types of skin cancer: basal cell carcinoma (BCC) and squamous cell carcinoma (SCC), collectively termed keratinocyte carcinomas, which are the most common (Lomas et al. 2012). In Australia, for example, essentially all keratinocyte cancers are attributable to high ambient UVR levels (Olsen et al. 2015). Melanoma develops from melanocytes and, though less common than other skin cancers, is responsible for most skin cancer mortality. Worldwide, melanoma was responsible for 59,782 global deaths (95% CI 47,602–72,671) with an age-standardized rate of one death per 100,000 persons (95% CI 0_7-1) (Karimkhani et al. 2017). An estimated 7,220 melanomas, or 63% of all cutaneous melanomas occurring in Australia in 2010, were attributable to high ambient UVR (Olsen et al. 2015).

Notably, in many predominantly fair-skinned populations, melanoma incidence is rising faster than most other cancer types (Erdei and Torres 2010), and numbers of cases are expected to continue rising over the next 15 y (Whiteman et al. 2016). There is some suggestion that increasing recreational and intentional UVR exposure is implicated in the rising incidence of melanoma, particularly in younger individuals (Elwood and Jopson 1997; Dal et al. 2007). The temporal trends of keratinocyte cancer incidence are difficult to determine because of unreliable registration data.

#### Ocular disease

Solar UVR causes various ocular diseases including eyelid malignancies (BCC and SCC), photokeratitis, pterygium and cortical cataract (Yam and Kwok 2014). In particular, UVB is implicated in cataract induction (McCarty and Taylor 2002), which is the most common cause of blindness globally (Resnikoff et al. 2004; Pascolini and Marriotti 2012). Cortical cataracts are more prevalent at lower latitudes where UVR is abundant (Javitt and Taylor 1994–1995; Sasaki et al. 2003). In experimental studies, a single corneal exposure to UV light can lead to detectable cellular and molecular changes (Delic et al. 2017). There are multiple risk factors for cataracts, but sunlight exposure is estimated to cause 10–20% of the cataract burden (Lucas et al. 2006; McCarty et al. 2000).

### Evidence for specific UV index cut-off values for protective measures

For the sake of clarity and simplicity in public health messages, current guidance on UV Index usage describes a stepwise implementation of preventive measures with no specific action required for a UV Index below 3; protection recommended for UV Index of 3 and above; and extra or reinforced protective measures at UV Index values of 8 and above. The 2015 Workshop reviewed the current evidence on the UV dose-response relationship, and specifically data to examine the concept of risk thresholds. Discussions of health risks from low UV exposures (<3) were balanced by consideration of potential Vitamin D deficiency.

Though the UV Index is based on risk of erythema, there is a body of research in humans and in immunocompromised animals suggesting that genetic damage, skin cancer, or immunosuppression may occur in response to lower doses of UV than are required to cause erythema (Rebel et al. 2005). For example, chronic exposure of mice with deficient DNA repair mechanisms to sub-erythemal doses of UVR (0.08 MED d^−1^) appeared sufficient to induce signature p53 mutations and squamous cell carcinoma (Rebel et al. 2005).

In human studies, photoimmunosuppression and UV-signature gene mutations have been used as surrogate markers for cancer risk. At sub-erythemal UV doses, molecular lesions [cyclobutane pyrimidine dimers (CPD)] have been reported in human skin after exposure to either UVA or UVB at 50 or 0.05 Jm^−2^, respectively (Mouret et al. 2006). Not all DNA damage leads to cancers: in immunocompetent individuals, such molecular lesions are usually cleared rapidly and so do not progress to fixed mutations and carcinogenesis. Using contact hypersensitivity models in humans and mice, local and systemic immunosuppressive effects of UVR that are evident at sub-erythemal levels of exposure have also been demonstrated (Poon et al. 2005; Byrne et al. 2002). As in other studies of the biological effects of UVR, these responses appear to be dose-dependent (Halliday and Lyons 2008).

These studies suggest that there is a broadly linear dose response to UVR in terms of DNA damage and immunosuppression and that the UVI scale represents a continuous risk gradient without specific risk thresholds. It appears also that UVR may have some measurable biological effects in fair-skinned individuals even in settings when the UVI is <3, given sufficient duration and frequency of exposure. There is, however, little or no epidemiological data to qualify or quantify the relative importance of sub-inflammatory versus erythemal doses of sunlight in causing skin cancer in humans.

While the adverse effects of overexposure to UVR are well documented, some UVB exposure is required for induction of synthesis of vitamin D, which is essential for musculo-skeletal health, and plays a potentially broader role in general health (Cranney et al. 2008; Chung et al. 2009). The precise amount of UVR exposure that minimizes cancer risk while preventing vitamin D deficiency is unknown but will vary by age, gender, diet, and skin type. It has been shown that adequate Vitamin D production can be achieved without erythema with short sun exposures at a UV Index of 6 (McKenzie et al. 2011) and that repeated exposure of fair-skinned individuals to UVR replicating 13-17 min of UK midday summer sunlight boosted 25(OH)D to sufficient levels (Felton et al. 2016). DNA lesions (CPD) were detected but were cleared by DNA repair mechanisms within 48 hours and did not accumulate. A darker skinned cohort in the same study had less DNA damage and smaller increases in vitamin D. On the basis of such evidence, Australia updated its position on the benefits and risks of solar UVR in 2016 stating that sensible sun protection in the Australian context does not put people at risk of vitamin D deficiency (Cancer Council Australia 2016).

### Evidence for the effectiveness of protective measures

Depending on the strength of the UV Index, several protective interventions are recommended in the form of physical barriers (shade, hat, clothes and sunglasses), exposure avoidance, and topical physical or chemical barriers (sunscreen). The workshop prioritized an assessment of sunscreens and sunglasses given the resource considerations involved with widespread implementation of sunscreen and sunglass recommendations.

#### Sunscreens

The workshop considered the literature regarding the effectiveness of sunscreen as a preventive agent for melanoma, BCC and SCC. Sunscreens are effective at preventing or delaying erythema from UVR exposure. That sunscreens can mitigate the risk of UV-related carcinogenesis is supported by a systematic review of molecular studies demonstrating their effectiveness in preventing UV-specific DNA damage in skin cells (Olsen et al. 2017) with one specifically relevant to melanoma (Hacker et al. 2013).

Though sunscreens effectively prevent sunburn and UV-related DNA damage, there is surprisingly little clinical trial data on their effectiveness in prevention of skin cancers (Lazovich et al. 2012). Four observational studies showed mixed protective and harmful associations between regular sunscreen use and melanoma risk; two found no association, one found a protective effect, and one found harmful effects (Whiteman et al. 1997; Autier et al. 1997; Youl et al. 2002; Lazovich et al. 2011). In the single randomized controlled trial with long-term follow-up, regular use of sunscreen reduced the observed rate of melanoma (Hazard Ratio 0.50; 95% CI, 0.24 to 1.02) and SCC (Rate Ratio 0.65, 0.45–0.94) but not BCC (Rate Ratio of 1.02, 0.78–1.35) (van der Pols et al. 2006; Green et al. 2011, 1999). Simulation studies (Olsen et al. 2015) estimated that regular use of SPF 15+ sunscreen prevented 1,729 melanoma (14% of melanomas) and 14,192 SCC (9% of SCC’s) in the Australian population in 2010. A recent cohort study (Ghiasvand et al. 2016) also found that the regular use of SPF 15+ and above reduced the melanoma risk.

#### Sunglasses

UV exposure of the eyes can be direct or reflected. While a substantial portion of direct ocular exposure can be shielded by a broad-brimmed hat, for both direct and reflected light, suitably manufactured sunglasses such as wrap-around sunglasses in particular are able to block most UV light at wavelengths below 400 nm from reaching the eyes. The spectral effect of UV exposure on the eyes has been measured in humans and animal models and is greatest at wavelengths between 270 and 320 nm (Pitts et al. 1977; Oriowo et al. 2002). UV-protective sunglasses (e.g., UV 400) are readily available that filter 99-100% of UV at these wavelengths.

Although international safety standards address the same broad performance requirements for sunglasses, there are notable differences in the variation in UVR transmittance criteria. The International Standard (ISO 12312), the United States standard (ANSI Z80.3), and the European standard (EN 1836) apply a transmittance protection rating up to 380 nm. The Australian and New Zealand standard (AS/NZS 1067) and the South African Standard (SANS 1644) require a stricter transmittance of up to 400 nm as defined by ICNIRP (ICNIRP 2004). Standards for manufacture and sale of sunglasses may not exist or be applied in low- and middle-income countries (Bazzazi et al. 2015). Unfortunately, to date there are no clinical trials demonstrating the effectiveness of sunglasses in cataract prevention to support the common-sense guidance on the use of sunglasses in the Global Solar UV Index guidance.

In addition to the recent systematic review assessing sunscreen and prevention of cutaneous DNA damage (Olsen et al. 2017), the U.S. Preventive Services Task Force (Lin et al. 2011) systematically reviewed behavioral counseling for primary prevention of skin cancer and found that primary care-relevant counseling could increase sun-protective behavior and decrease indoor tanning.

### Issues in implementation of the UV index and related guidance

#### Local adaptation of guidance

The UV Index scale was originally developed in Canada, and the current guidance on the use of the UV Index is oriented toward fair-skinned populations because they are at the greatest risk of skin cancer. UV exposure may be much higher in other regions, but the cancer risk is lower because of melanin protection of the skin of inhabitants. For example, the levels of UVR measured in the Altiplano Region of Bolivia as well as in other high altitude locations in South America exhibit extreme peak values of the UV Index exceeding 15 for a third of days per year. The incidence of melanoma in Bolivia is estimated to be low, around 2.5 per 100,000 persons, based on data from neighboring countries (Ferlay et al. 2012). However, skin pigmentation varies considerably in Bolivia, and knowledge of skin cancer incidence rates in different ethnic groups is lacking.

Similarly, in South Africa the UV Index reaches 12–14 in summer. In the black-skinned population, the incidence of melanoma in the period 2000-2004 was low at 1.0 for males and 1.2 for females per 100,000, while in the fair-skinned population the incidence was more than 15 times higher at 20.5 for males and 16.5 for females (Norval et al. 2014). Because the same UVR exposure will result in much less vitamin D production and much reduced DNA damage in brown-skinned individuals, it is sensible that guidance for preventive measures be adapted by countries to meet the needs of their populations (Farrar et al. 2013).

A proposal for modification of the UV Index was made to enhance its applicability to large regions of the world with higher levels of UVR exposure and more darkly pigmented populations (Zaratti et al. 2014). In particular, it was suggested that the numerical scale of the UV Index as represented graphically be extended to higher values and that health messages be tailored to regions, skin types and durations of exposure. These health messages are likely to be most useful if generated and broadcast locally rather than included in a global document primarily aimed at combatting the rising rate of skin cancer in high-risk populations. As stated in the current Global Solar UV Index Practical Guide, “The basic scheme for UV Index reporting and sun protection can be varied and expanded through the use of additional messages at the national or local level.”

Fixed UV Index values for defining risk and associated preventive measures are unlikely to be useful in settings where there is considerable diversity of ethnicity. More complex and more accurate ways to predict personal risk based on UVI, skin type, and duration of exposure are possible, and it is hoped predictions might be developed based on experimental studies identifying thresholds of exposure that boost vitamin D production without accumulation of molecular damage. The availability of portable UV monitors, if sufficiently accurate, and mobile smart-phone applications may be useful for individuals to manage their sun exposure. Further research was also called for investigating the effects of UVR exposure in immunocompromised individuals given the high prevalence of HIV infection in some regions with intense sunlight.

### Use of the UV index as a public health awareness tool

The objective of the UV Index is to stimulate informed decision-making to mitigate risks by creating risk perception around the potential for skin damage. To be effective in raising awareness, the UV Index messaging must accurately and compellingly communicate risks that are of personal interest to the audience, and must provide clear, simple, and effective strategies. The current UV Index applies a three-tier approach for increasing awareness of hazardous UVR levels. A two-tiered approach as considered in the Cancer Council Australia Position Statement (Cancer Council Australia Position Statement 2016) with a “minimal to no risk” category, and a single “risk” category (>3) for which all protective measures are used, was considered as a simple call-to-action message but with the caveat that high numbers may not produce more motivation for increased protection. The current graded approach has the benefit of reinforcing the common-sense notion that greater radiation requires greater protection, and helps motivate behavioral change.

To date, communication around the UV Index—mostly through radio, television, and mobile devices—has had mixed success. In Canada, about 20% of people routinely consult the UV Index before going outside for an extended period, and more than 60% of people use additional protection when the UVI is high. Unfortunately, people in many countries still do not know the difference between heat and UVR (Wong et al. 2015). In a German survey (Boerner et al. 2010), the UV Index had poor recognition with only 30% having ever heard or read anything about the UV Index – a similar recognition frequency was found among school children in South Africa (Wright et al. 2015a). Interestingly, broadcasting of the UV Index in South Africa was dropped after 10 y because of poor media uptake and the monotony of unchanging UVI values in that climate. In Sweden and other Nordic countries, where the incidence of melanoma is high, stimulating the community with UV Index broadcasts is challenging when the UV Index remains below 3 in winter and varies little during summer. In Denmark, a cross-sectional study showed that 92% of the Danes had heard of the UV Index for protection, but there was no association between having heard of the UV Index and sun protection behavior (Bentzen et al. 2013). Sunscreen is made available free of charge to South Africans with oculo-cutaneous albinism, but is not well taken up (Wright et al. 2015b). In South Africa, it is a regular practice for people to turn their backs to the sun (Wright et al. 2015b).

The UV Index cannot function alone as an effective means to change behavior. It needs to be part of a much broader, integrated strategy on sun protection. There is some evidence that a partnership approach that integrates the UV Index into a combination of individual-directed strategies, community action, mass media campaigns, and advocacy tools may enhance utility (Shih et al. 2009; Saraiya et al. 2004). Programs based on various health behavior theories that have used this approach have been able to provide effective programs that change people’s attitudes and intentions. Theories invoked have included the preventive health model, the social cognitive model, and the health belief model (Janda and Green 2014). One such program is SunSmart, a campaign that has prevented 43,000 skin cancers between 1988 and 2010 in Victoria, Australia (Shih et al. 2017). Volkov et al. (2013) also found some evidence of improvement in use of sun protection and a decrease in sunburn, but further improvements were required. Knowledge about the effectiveness of the UVI as a risk communication tool remains thin (Italia and Rehfuess 2011; Allinson et al. 2012), especially in developing countries where there are many competing health priorities.

Discussions on modifying the guidance on the use of UV Index reflected competing interests. The desire for policies based on solid scientific evidence militates against the use of simple thresholds of exposure risk. The desire for inclusivity and global use drives consideration of extending the UVI scale to higher values and for including skin type and duration of exposure in the UVI guidance. In competition with desire for greater global inclusivity of the UVI guidance is the finding that among high-risk populations for skin cancer, the messaging is already felt to be complex (11 scalar points, five risk categories, three levels of risk mitigation) and often confused with other conflicting messages (e.g., time to burn, SPF recommendations). At the workshop, there were some indications that the UV Index awareness was increasing in settings that have an established public health approach; however, the understanding in low-income countries remains poor.

## CONCLUSION

The 2015 UV Index meeting in Melbourne, Australia, supported many of the conclusions of the 2013 meeting in Germany: awareness of the need for protection at UV Index of 3 and above is still of high public health relevance. While the UV Index is not a stand-alone tool, it can assist in combination with broader sun protection strategies. A number of new scientific publications have emerged since 2013 that provide indications of progress on relevant research questions, but the human evidence remains too limited to justify a modification of the WHO Global Solar UV Index and its guidance.

The UV Index continues to be a useful standardized tool to estimate risk of harmful solar UVR exposure in many countries and can be integrated with additional messages at the national or local level. Further research is needed to improve the effectiveness of the UV Index as a public awareness tool. It is possible that modern technology, such as UV Index messages delivered to mobile devices, can provide this information more widely in the population. However, the final goal of improving sun protection behavior will only be attained by delivering comprehensive community-wide health promotion interventions.
